# Duodenal stenosis in adult malrotation: When Ladd procedure is not enough: A case report

**DOI:** 10.1016/j.ijscr.2024.109713

**Published:** 2024-04-30

**Authors:** Ebenezer Akomea-Agyin, Kwabena Agbedinu, Charles Kofi Dally, Fareeda Galley, Emmanuel Osei Kankam, Gabriel Edudzi Banini

**Affiliations:** a Komfo Anokye Teaching Hospital, Directorate of Surgery, P.O. Box 1934, Kumasi, Ghana; b Komfo Anokye Teaching Hospital, Pediatric Surgery Unit, P.O. Box 1934, Kumasi, Ghana

**Keywords:** Adult malrotation, Ladd bands, Intermittent midgut volvulus, Duodenal stenosis, Heineke-Mikulicz strictureplasty, Case report

## Abstract

**Introduction:**

Congenital causes of duodenal obstruction can be grouped into intrinsic and extrinsic causes. The degree of obstruction caused by such etiologies determines the severity and timing of symptom presentation. Early neonatal diagnosis is common in patients with etiologies that present with high degrees of obstruction such as atresia whereas etiologies that cause lesser degrees of obstruction such as malrotation and duodenal stenosis can go undiagnosed into adulthood.

**Presentation of case:**

We report a case of a 24-year-old female who presented with acute on chronic abdominal pain with bilious vomiting. She was diagnosed with intermittent small bowel volvulus which resolved spontaneously but was found to have adult intestinal malrotation diagnosed intraoperatively. She had a Ladd procedure done but had persistent obstructive small bowel symptoms after the Ladd procedure. She was found to have duodenal stenosis from fibrosis of the duodenum on relaparotomy which was treated surgically with Heineke-Mikulicz strictureplasty leading to total resolution of symptoms.

**Discussion:**

Congenital extrinsic and intrinsic causes of partial duodenal obstruction such as Ladd bands in malrotation and duodenal stenosis respectively, can co-exist and persist into adulthood due to their lesser symptomatology and degree of obstruction. Surgical treatment must identify and correct both conditions when they co-exist to ensure complete resolution of symptoms.

**Conclusion:**

This case report highlights the association of duodenal stenosis with adult malrotation which may account for persistent symptoms after the Ladd procedure and suggests the use of Heineke-Mikulicz strictureplasty as a complementary procedure for complete symptom resolution.

## Highlights

• Gastrointestinal malrotation can present concomitantly with duodenal stenosis.
• Both conditions can persist isolated or concomitantly undiagnosed into adulthood due to their vague symptomatology.
• Surgical management of malrotation in the presence of duodenal stenosis requires other surgical interventions.
• Heineke-Mikulicz strictureplasty is a simple and add-on procedure to Ladd procedure in treating duodenal stenosis in malrotation.
• A high index of suspicion is required to identify duodenal stenosis in the presence of gastrointestinal malrotation especially in adults.

## 1 Introduction

Congenital duodenal obstruction is a common presentation in the neonatal group. The causes can be extrinsic or intrinsic. The extrinsic factors include Ladd bands in malrotation, annular pancreas, anterior portal vein, and duodenal duplication. The intrinsic factors include duodenal atresia, duodenal webs and duodenal stenosis [1,2]. These conditions are mainly reported in neonatal groups due to their early symptomatology. However, these symptoms depend on the degree of obstruction [2]. Higher degrees of obstruction prompt early recognition as early as prenatal life. Lesser degrees of obstruction, however, may persist into adulthood without a high index of suspicion [3,4]. Common causes of duodenal obstruction that can present with lesser degrees of obstruction are Ladd bands in malrotation and duodenal stenosis [3].

We present a rare case of congenital duodenal obstruction with combined intrinsic and extrinsic causes, namely, duodenal stenosis with gastrointestinal malrotation persisting into adulthood. This case report has been reported in line with the SCARE Criteria [5].

## 2 Case report

The patient was a 24-year-old tertiary-educated female who worked in a remote village as a teacher. The patient presented to our emergency department via ambulance as a referral from a hospital where she had been managed for two days for pelvic inflammatory disease (PID). She presented with a 2-day history of sudden onset central abdominal pain that was piercing, constant, aggravated by walking or lying flat but relieved on bending over. It was not associated with food intake or high-fat meals but with fever, chills, headaches, nausea, and non-bilious vomiting that later became bilious. The pain was rated 9/10 and rendered her unable to perform her daily activities. Her past medical history revealed chronic recurrent abdominal pain that was associated with vomiting for the past seven years. It was managed as either peptic ulcer disease (PUD) or gastroesophageal reflux disease (GERD) with omeprazole without a confirmatory test. She had no prior surgeries. She did not smoke cigarettes, drink alcohol, or use illicit drugs. She was single and sexually active. She was triaged and managed from the emergency department as a gynecological case with suspicion of PID. Our general surgery team was consulted after she experienced her first episode of bilious vomiting, on suspicion of intestinal obstruction on admission day three.

The examination revealed a young female adult who was met bent forward by her bed moaning in pain. On lying down she assumed a knee-chest position. She was neither pale nor jaundiced. Her temperature was 37.4 degrees Celsius. She had tachycardia of 105 beats per minute (bpm) and a blood pressure reading of 110/68 millimeters of mercury (mm Hg). The precordium examination had normal findings. She had tachypnea of 30 cycles per minute (cpm), and air entry was normal with vesicular breath sounds throughout the lung fields. The abdominal examination revealed a flat abdomen that moved with respiration. No scars were seen on her abdomen. She had generalized abdominal tenderness marked in the epigastric and umbilical areas with guarding and rebound tenderness. There was no organomegaly palpated. Digital rectal examination was normal with normal soft stools in the rectal ampulla.

Laboratory investigations showed anemia (HB: 9.1 g/dL) with normal blood urea, creatinine, electrolytes, and liver function tests (See Table 1).

**Table 1:** Laboratory investigations prior to first surgical intervention.

	Parameters	Value	Reference range
Complete blood count (CBC)	Hemoglobin	9.1 g/dL	11.5–16.0
	Hematocrit	29.0 %	34.7–46.0
	Platelet count	210 × 10^3^/μL	140–440
	White blood cell count	8.59	4.00–10.00
Serum biochemistry	Albumin	46.7 g/L	35.0–52.0
	Globulin	26.6 U/L	25.0–35.0
	Aspartate Aminotransferase (AST)	23 U/L	0–40
	Alanine Aminotransferase (ALT)	17.0 U/L	0–41
	Gamma Glutamyl transferase (GGT)	13.7 U/L	6.0–71.0
	Alkaline Phosphatase (ALP)	68.0 U/L	35–129
	Creatinine	69 μmol/L	44–106
	Urea	2.53 mmol/L	2.10–8.30
	Serum amylase	125.90 U/L	25–104
	Serum lipase	95.00 U/L	23–300
Serum electrolytes	Potassium	3.7 mmol/L	3.50–5.10
	Sodium	134 mmol/L	135–145
	Chloride	106 mmol/L	97.0–110.0

She was kept nil-per-os (NPO). A nasogastric (NG) tube decompression was done with about 450mLs of bilious fluid drained, after which the abdominal pain remarkably subsided to a pain score of 2/10. She was managed on intravenous (IV) fluids and medications (lactated ringers solution, normal saline solution, dextrose 5 % solution, omeprazole, morphine, and paracetamol). An impression of acute intestinal obstruction was made with a differential of acute pancreatitis. Further investigations revealed normal serum amylase, and lipase levels (See Table 1).

**Fig. 1:**
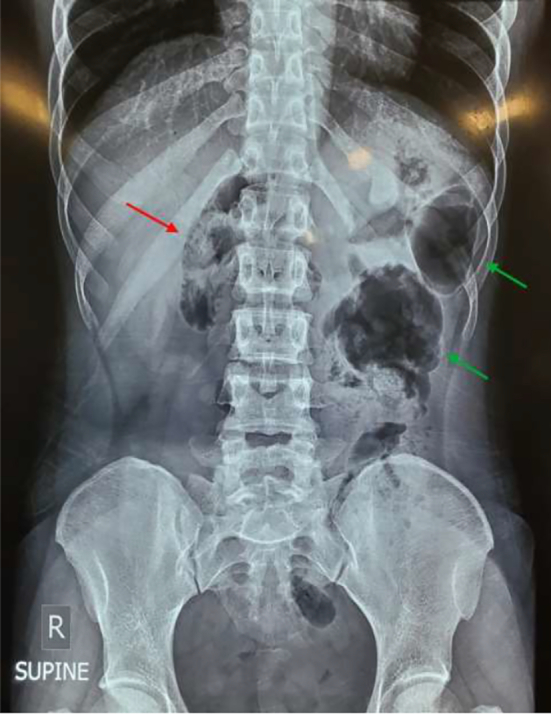
Supine plain abdominal X-ray showing caecal shadow in epigastrium (red arrow), and large bowel loops localized on the anatomical left (green arrows). (For interpretation of the references to color in this figure legend, the reader is referred to the web version of this article.)

An abdominal x-ray was considered normal under the consideration of intestinal obstruction. A review of the x-ray in hindsight, post-surgery, revealed the absence of a caecal gas shadow in the anatomical right lower quadrant. It was noted in the epigastrium with the large intestinal loops seen on the left side of the midline on the X-ray (Fig. 1).

An abdominal ultrasound showed normal findings. A computed tomography (CT) scan was done which reported swirling of the vessels and mesentery in the central abdomen around the axis of the superior mesenteric artery (SMA) with moderate distension of the central loops of the small bowel. This was interpreted as a midgut volvulus with small bowel obstruction.

**Fig. 2:**
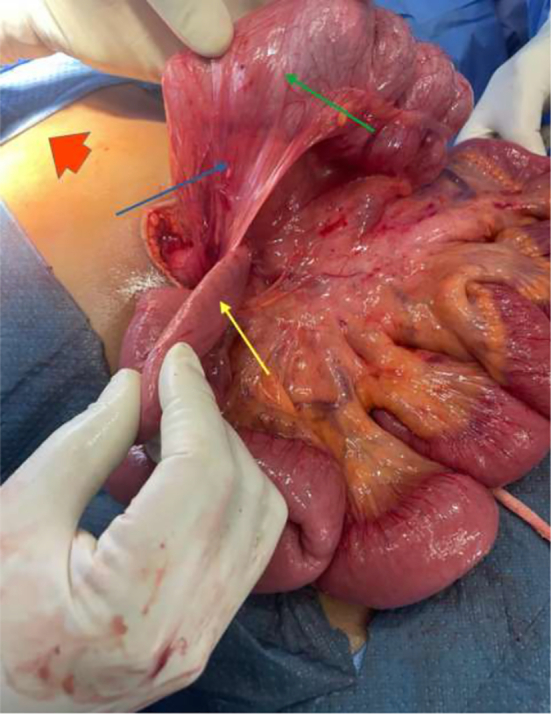
Intraoperative findings showing Ladd bands (blue arrow) arising from a high riding caecum (green arrow) and crossing a segment of duodenum (yellow arrow). The red arrow points to the head end of the patient. (For interpretation of the references to color in this figure legend, the reader is referred to the web version of this article.)

Based on these findings an emergency exploratory laparotomy was performed by a specialist and a resident in general surgery on account of the small intestinal volvulus about 2 h after receiving the CT scan report. The intra-operative findings were a high-riding caecum and Ladd bands constricting a segment of the duodenum on the right side (Fig. 2). The ligament of Treitz was on the right side of the midline. These were indicative of malrotation. The bowel was viable, and no volvulus was noted. A Ladd procedure was done; the Ladd bands were divided, and the C-loop of the duodenum was straightened. The adhesions surrounding the SMA were divided, and a prophylactic appendectomy was performed. The caecum and colon were mobilized and reflected to the left with the small intestines to the right in a “non-rotational” position.

The patient recovered uneventfully up to postoperative day five when she experienced colicky abdominal pain after meals. The pain was associated with a single episode of vomiting containing previously eaten food but progressed to several episodes of bilious vomiting. An NG tube was inserted which immediately drained about 480mLs of bilious gastric content. She recorded a blood pressure of 144/96 mm Hg, a pulse of 73 bpm, a respiratory rate of 20 cpm, and an oxygen saturation of 99 % on room air. An electrocardiogram and echocardiogram revealed hypertrophic heart disease. Serum electrolytes and renal function tests remained normal (Table 2). She also developed surgical site infection with a culture and sensitivity results indicative of enterobacter aerogenes with sensitivity to amikacin. The patient was managed NPO and given intravenous fluids, IV antibiotics including IV amikacin, IV antihypertensives, IV omeprazole, IV analgesics, and placed on subcutaneous clexane.

**Table 2:** Laboratory investigations after first surgical intervention and prior to second surgical intervention.

	Parameters	Value	Reference range
Complete blood count (CBC)	Hemoglobin	10.2 g/dL	11.5–16.0
	Hematocrit	29.2 %	34.7–46.0
	Platelet count	391 × 10^3^/μL	140–440
	White blood cell count	8.57	4.00–10.00
Serum biochemistry	Creatinine	55 μmol/L	44–106
	Urea	1.29 mmol/L	2.10–8.30
Serum electrolytes	Potassium	3.2 mmol/L	3.50–5.10
	Sodium	135.0 mmol/L	135–145
	Chloride	97.0 mmol/L	97.0–110.0

**Fig. 3:**
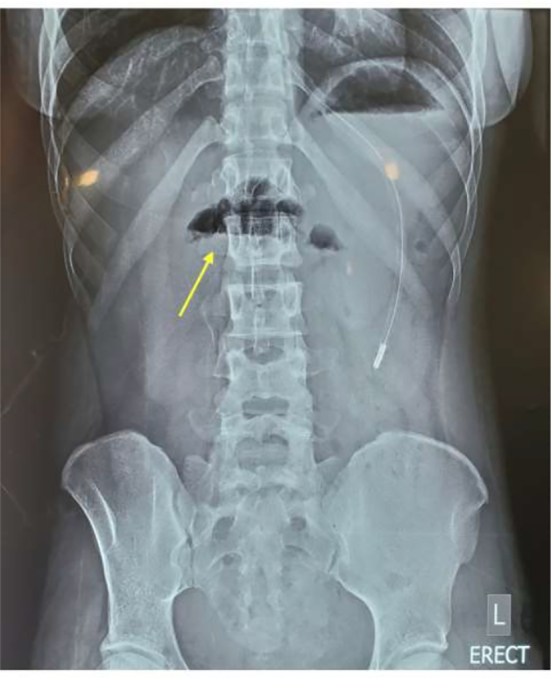
Plain erect abdominal X-ray showing air fluid level indicative of the double-bubble sign (yellow arrow). (For interpretation of the references to color in this figure legend, the reader is referred to the web version of this article.)

**Fig. 4:**
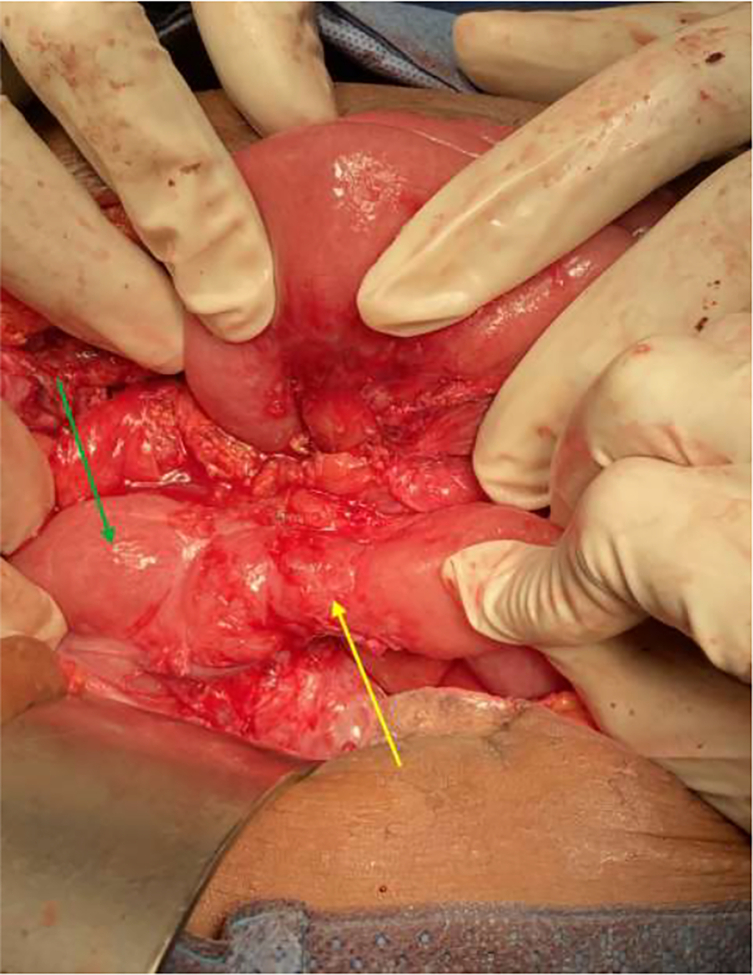
Intraoperative finding of stenotic duodenal segment (yellow arrow) with dilatation of its proximal end (green arrow). (For interpretation of the references to color in this figure legend, the reader is referred to the web version of this article.)

**Fig. 5:**
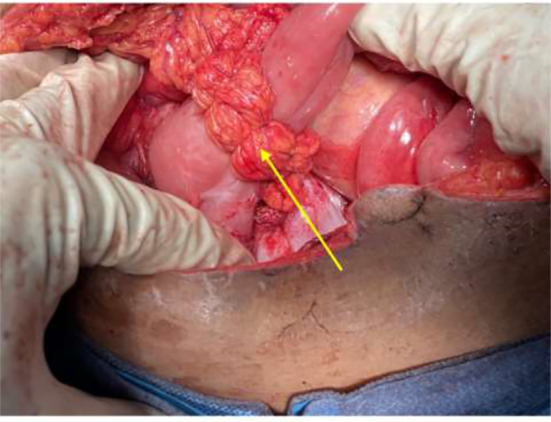
Intraoperative picture showing omental patch reinforcement (yellow arrow) of Heineke-Mikulicz strictureplasty. (For interpretation of the references to color in this figure legend, the reader is referred to the web version of this article.)

The pediatric surgery team was consulted due to their expertise in managing malrotation. They advised watchful waiting for delayed gastric emptying that may result after the Ladd procedure with a differential of incomplete Ladd band release and duodenal stenosis from the constriction of the duodenum seen in the first surgery. The patient was managed conservatively and optimized to postoperative day 12 when she was stable. A repeat abdominal x-ray showed the double bubble sign on the erect view (Fig. 3). A decision was taken with the pediatric surgery team to have a relaparotomy done. An informed consent was taken from the patient and surgery was performed by a specialist in pediatric surgery and a resident in general surgery under direct supervision of a consultant. The intraoperative findings were duodenal stenosis from a fibrous constriction of the duodenum with few non-constricting adhesion bands holding down the duodenum. The duodenum was dilated proximal to the stenotic end (Fig. 4). The bands were released to free the duodenum completely. Duodenal patency was tested with saline injected into the stomach and milked down through the duodenum. The duodenum was stenotic. A Heineke-Mikulicz strictureplasty was done. The patency of the duodenum was confirmed, and a leak test was done to confirm the integrity of the anastomosis. An omental patch was added to reinforce the anastomosis (Fig. 5).

The patient on postoperative day six experienced non-bilious vomiting after feeding. She was managed as delayed gastric emptying. She reverted to light diets and graduated to full feeds over three days, and the vomiting resolved. She was switched to oral medications and discharged on postoperative day ten after the second surgery in a stable condition.

The patient was seen on an outpatient basis with normal laboratory investigation. She complained of occasional colicky epigastric abdominal pain that resolved over a 2-month period during her outpatient care with conservative treatment. The patient is currently pain-free, has not experienced any episodes of vomiting as before surgery, has a normal bowel habit, and has returned to work as a teacher.

## 3 Discussion

Malrotation in adults can present as an emergency. The most important cause is a secondary midgut volvulus which presents with bilious vomiting, fever, tachycardia, and generalized tenderness on abdominal examination [6]. The patient presented with the above signs and symptoms.

The presentation of midgut volvulus in malrotation can be either ‘malrotation with volvulus’ or ‘malrotation with partial or intermittent volvulus’ [7]. Malrotation in adults can also present as chronic gastrointestinal symptoms marked mainly by abdominal pain and vomiting [6,8,9]. About a third of these patients would have been misdiagnosed with GERD in the past [9]. The patient had been misdiagnosed as having PUD or GERD and omeprazole did not relieve the pain. We believe the acute presentation was due to a secondary intermittent midgut volvulus as described by Langer [7].

Preoperative diagnosis of malrotation in adults is difficult. The diagnosis is usually intraoperative [6]. Imaging diagnosis in adults usually requires multiple imaging modalities. The gold standard of diagnosis in all age groups remains the upper gastrointestinal series though CT scan is becoming the imaging of choice in adult patients [8]. A plain abdominal X-ray may show signs of abnormally placed bowel with the large bowel markings usually on the left of the midline and the small bowel markings on the right of the midline as was seen in this patient. CT scan comes in handier in adult populations in the setting of midgut volvulus where diagnosis is made about 67 % of the time. It shows the whirl or whirlpool sign, which is the swirling appearance of the SMV, and mesentery twisted around the axis of the SMA [10,11]. The midgut volvulus was diagnosed via CT scan showing the whirl sign.

The mainstay of treatment in an emergency setting is surgery. The volvulus is reduced and if non-viable resected. The underlying malrotation is treated with the Ladd procedure. The effectiveness of the Ladd procedure in symptom resolution is estimated at 89 % [8]. The Ladd procedure improved the acute symptoms of the patient, but the intermittent abdominal pain and vomiting persisted after postoperative day five.

Duodenal stenosis is a rarely reported intrinsic cause of duodenal obstruction either as an isolated entity or in association with gastrointestinal malrotation. The diagnosis is a challenge because it presents with the indolent symptoms of partial obstruction [4]. Duodenal stenosis is diagnosed on X-rays with the presence of a double-bubble sign with distal air [1,2,4]. It is important to note that duodenal obstruction presenting concomitantly with malrotation is easy to miss intraoperatively if the surgeon is not looking for it. When suspected, it is advised the surgeon milks gastric content from the proximal to the distal duodenum intraoperatively to confirm the diagnosis [7]. The procedure of choice in duodenal stenosis is a surgical bypass of the stenotic segment with a duodeno-duodenostomy with or without a post-anastomotic jejunostomy feeding [2,4]. In the first surgery, the segment of the duodenum was not tested for patency after the Ladd procedure. During the second surgery, patency was tested by instilling saline into the stomach and milking it down the duodenum. This revealed the stenosis. A Heineke-Mikulicz strictureplasty with an omental patch done, restored duodenal patency.

## 4 Conclusion

Duodenal stenosis and Ladd bands in malrotation can go undiagnosed even into adulthood and can exist concomitantly in a patient with partial duodenal obstruction. We believe intrinsic duodenal stenosis may contribute to the failure rate of the Ladd procedure in malrotation. We advise the routine milking of gastric content through the duodenum to establish its patency after the Ladd procedure. We propose Heineke-Mikulicz strictureplasty as a simple alternative to the surgical treatment of duodenal stenosis in adult patients.

## Consent

Written informed consent was obtained from the patient for publication of this case report and accompanying images. A copy of the written consent is available for review by the Editor-in-Chief of this journal on request.

## Ethical approval

Ethical approval was waived at our institution. The ethical review waives ethical approval of case reports as patient identification is not revealed and would not be harmful to the patient.

## Funding

This research did not receive any specific grant from funding agencies in the public, commercial, or not-for-profit sectors.

## CRediT authorship contribution statement

All authors contributed to the present case report.

Ebenezer Akomea-Agyin: Conceptualization, Data Curation, Writing - Original draft preparation, Writing - Review and Editing, Resources and participated in patient management. Kwabena Agbedinu: Writing - Review and Editing, Validation, Resources and participated in patient management. Charles Kofi Dally: Writing - Review and Editing, Supervision, Resources and participated in patient management. Fareeda Galley: Resources and participated in patient management. Emmanuel Osei Kankam: Resources and participated in patient management. Gabriel Edudzi Banini: Resources and participated in patient management.

## Declaration of competing interest

The authors declare that they have no known competing financial interests or personal relationships that could have appeared to influence the work reported in this paper.
